# Clinical Lectures on Hypertrophy of the Prostate with Retention of Urine

**Published:** 1855-12

**Authors:** John Adams

**Affiliations:** Surgeon of the Hospital


### SELECTIONS.

*Clinical Lectures on Hypertrophy of the Prostrate with Retention of Urine.* Delivered at the London Hospital, by JOHN ADAMS, Esq., F. R. C. S., Surgeon of the Hospital.

GENTLEMEN: These cases of prostatic hypertrophy are of every day occurence. Their importance may be approximately estimated by their frequency, and the disastrous consequences to which they too often lead. I am, therefore, glad to have the opportunity of directing your attention to-day to this subject.

We have received into this hospital during the last year 177 cases of retention of uterine, from all causes. Of these, only six have happened to females—the rest to males. The reason of this vast disproportion is obvious enough. The length of the urethra in the male, the existence of various muscles surrounding it, the position of the prostrate gland at its commencement, the lodgement sometimes of small calculi in various parts of it, the existence of stricture, (which is a disease almost exclusively of the male urethra,) the severe attacks of inflammation to which the urethra is liable in gonorrhœa, all these causes predispose the male subject to retention of urine, and explain the important fact alluded to. I may also mention the injuries to which the male urethra is liable; all of which lead to a similar result. Now, I think I may venture to assert that, of all these sources of obstruction, hypertrophy of the prostrate gland is the most common cause of the retentions of urine which are brought to this hospital. I regret that the statistics of the hospital do not give us information on this subject; but in future, a better system of registry, now about to be adopted, will supply the omission.

This enormous mass of cases, which almost invariably come under your own immediate inspection and treatment, (independent of the surgeon,) give you such a field of practical experience as cannot be obtained anywhere but at institutions like this. I am sure you must have remarked that it is my invariable custom to habituate the dressers of this hospital to the introduction of the catheter in all cases; and even in the worst cases of stricture, requiring the nicest manipulation, if I find that a student under my direction succeeds in the passing of an instrument into the bladder, I confide the case at once to him to bring it to a successful termination. I dare say, you remember my saying to one of the students in the beginning of the present session, in allusion to a case of retention of urine, that the best clinical instruction I could give, was to teach him to pass a catheter. The consequence of all this is, that the surgeon is seldom sent for to cases of retention. The house-pupils, under the eye of the house-surgeon, have generally done the business before his usual visit. It is, of course, not always so, as the present case exemplifies.

Let me now consider the case before us. Here is a case of retention of urine occurring in an old man of sixty,seven, who was the subject of a similar affection about a fortnight ago. He could not explain the cause, but says that he was called on to pass urine many times in the night, and at eight in the morning complete retention came on. The very simple fact of the age of the patient should at once arrest your attention, as it is a most important element in estimating the cause of the retention. An old woman slips upon the pavement and injures her hip, and cannot walk; she is brought to the hospital, and, without examination, you at once infer that she has fractured the neck of the thigh bone. So retention of urine, occurring in an old man, leads you to the conclusion that the retention arises from enlargement of the prostrate gland, and this is generally the case. Nevertheless, you ought to inquire into the history of the patients case, and he will tell you, perhaps, that he never had any complaint in these parts, or any difficulty in passing urine until during the last six months, when he has found that he has been called upon more frequently to pass his urine, especially during the night, and that the stream of urine, although sufficiently full, perhaps, has lost much of its force. Sometimes there has been a great deal of pain accompanying the efforts at expulsion. Well, with the brief history which you gather from your patient, your notion or idea is strengthened; but to set the question at rest you pass your finger per anum, and at once all doubt vanishes, as you feel the gland enlarged, possibly to the size of your fist. It is a curious fact that patients seldom experience any pain during the progress of the enlargement; but I have often ascertained that a slight tingling sensation on the dorsal aspect of the glans penis, in the course of the dorsal nerve, has been experienced, but no importance has ever been attached to it; in fact a man will say that he never knew he had any disease about him of the nature of enlargement at the neck of his bladder until informed of it by his surgeon. You have now ascertained the cause of the disease, and you proceed to act accordingly. There is no use in wasting your time, and exhausting your patient's strength, by leeching, purging, warm baths, &c.; the catheter is the *unicum remedium*, and to this you at once resort. You call for a prostatic catheter, place your patient at the edge of the bed, or in the erect position, introduce the instrument *secundem artem*, and draw the water off. You then take out the catheter, order the patient to bed, and anticipate the necessity of a re-introduction of the instrument in three or four hours. I say in three or four hours, for you will usually find that the water begins to pour down rapidly from the kidneys, and the bladder is refilled and requires, even more urgently than before, to be emptied; or possibly the passing of the catheter has ruptured some of the large veins in the neighborhood of the prostrate, and a very considerable quantity of blood has become mixed with the urine, to the great increase of the contents of the bladder. The bladder should be entirely emptied at each introduction of the catheter.

To proceed with the case: there was a difficulty which could not be overcome. The dresser of the previous week, under whose care he had been relieved, and who had recognized the case as one of enlarged prostrate, by examination per anum, was foiled. The prostatic catheter was passed up to the hilt, and the elastic catheter was used without effect. No very small instruments were used in the case. It now became my duty, at the usual visit, to examine the case myself, and to relieve the patient at all hazards. I was apprised of the nature of the case, and finding that the ordinary instruments had been unsuccessfully used I directed the patient to stand up, and took a full-sized elastic catheter, without stylet, straightened the instrument, and passed it along the urethra.

I felt it hitch against the prostrate, and having withdrawn it about an inch, I advanced it again, and it entered the bladder at once. Gentlemen, there is no mistaking the sensation experienced as the catheter enters the bladder. It goes in, as the Americans would say, *slick;* and I dare say you know the meaning of that term.

My object in the present lecture is to explain the reason why one instrument succeeds in one case and another in another. Mark me; I take no credit for the success of the manœuvre in this case. We are all liable to be foiled in our best endeavors; and I do not give much credit to those who say they are never foiled in passing a catheter. An inspection of the preparations preserved in all our museums, will show why their efforts have been crowned with success (such as it is.) Perforation of the prostrate stands in judgment against them. I cannot help thinking that after fumbling about a case for a long time our fingers get fatigued and refuse to obey our will or inclination, and this is a cause of failure. A fresh surgeon comes forward; he gains knowledge by the failing attempts of his predecessors; he takes the very instrument they *have not taken*, and, with those advantages, no wonder he succeeds. If you will take pains to examine the bodies of patients who have died of retention of urine, and dissect out the bladder and urethra, you will find that the prostrate enlarges in various directions. Sometimes it is the left lobe which is enlarged; sometimes it is the right; sometimes both are enlarged together, as is very generally found to be the case; but most frequently, the third or middle lobe is the seat of the enlargement, and by rising up at the orifice of the urethra, it dams up the bladder; indeed, John Hunter called it the valve of the urethra—and prevents the urine from escaping. Attention to these varied conditions will, I think, often explain why one instrument will pass more readily than another in particular cases. Thus the middle lobe being the seat of enlargement, and the cause of retention, if this body rises up equally, the urethra elongates to a great extent, and it requires first, a large, long catheter—long to traverse the entire length of the canal, and large to prevent it hitching in the prostatic sinus; next, that the point of the instrument should be tilted up, either by depressing the handle, or by inserting the finger per anum, so as to carry it well over the projecting third lobe; a gentle pushing forward of the catheter is then requisite, and the bladder is entered at once.

In other cases, the lateral lobes of the gland are unequally enlarged, the third lobe being free from any extraordinary increase of size, and the retention is due to the coaptation of the two lateral lobes, which sometimes dovetail into one another, and the urethra becomes deviously directed to one side or the other. In this case, the large silver prostatic catheter will not answer, and either a smaller instrument, or one with a small abrupt curve, (such as I now show you,) or, what I think is much better, a large elastic catheter, without a stylet, should be gently pushed along the urethra; it will gradually make its way through the winding channel and enter the bladder at once. I think, in the case before us, the prostrate has been enlarged in this latter method, and hence the facility with which the catheter was introduced. I am almost tempted in some cases to try a long, nearly straight, silver catheter, but of course this would not answer in cases of hypertrophy of the third lobe; but if this be enlarged, and inclined more to one side than the other, the elastic catheter, introduced as I have just stated, may make its way more readily and easily than even the large prostatic catheter. Sir Benjamin Brodie generally employs the elastic catheter; and there is an obvious advantage in its employment as a general rule—namely, that, independent of other considerations, it is more easily retained in the bladder, if you wish to keep it in.

Let us further examine the case before us, and apply what we have just said: it appears in the report that the old man passed his urine himself after the first day. Now, can we explain this? you may ask. Where is the enlargement to which the retention was attributed? Is it gone so rapidly? These questions no doubt suggest themselves, and are pertinent enough, and I offer the following explanation: There has been superadded to the hypertrophy of the prostrate, vascular congestion, and this having been relieved by the bleeding, occasioned by the catheter, and by the rest, purging, &c., the gland has been relieved, and has resumed its wonted size, (of course still much enlarged,) and the parts, therefore, have permitted the urine to escape. I can hardly think that anything like spasm of the prostrate exists, although it is a muscular organ, or that it can have produced the impediment; and if spasm of the urethral muscles were the cause, the catheter could not so readily have been passed. Venous congestion, too often, perhaps, overlooked, has, I think, been the immediate cause of the retention of urine. I know instances of cases which have led me to this conclusion. I knew an old gentleman who always suffered retention after a nocturnal emission. In this case one can hardly suspect any sudden increase of size of the gland, except such as may have resulted from vascular congestion.

I need only say, in conclusion, that the case before us, having been completely relieved by the use of the catheter, employed night and morning for several days, is discharged with the certainty that at some future time he will return to us, and require a similar mode of treatment; and so he will go on until the termination of his life, the intervals of retention being briefer and briefer, unless you put him in a way to do something for himself, of which I shall speak at another lecture.—*London Lancet.*

